# Ruthenium Chloride—Induced Oxidative Cyclization of Trans-Resveratrol to (±)-ε-Viniferin and Antimicrobial and Antibiofilm Activity Against *Streptococcus pneumoniae*

**DOI:** 10.3389/fphar.2019.00890

**Published:** 2019-08-14

**Authors:** Mukesh Kumar Yadav, Karabasappa Mailar, Jagadeesh Nagarajappa Masagalli, Sung-Won Chae, Jae-Jun Song, Won Jun Choi

**Affiliations:** ^1^Department of Otorhinolaryngology–Head and Neck Surgery, Korea University Guro Hospital, Seoul, South Korea; ^2^Institute for Medical Device Clinical Trials, Korea University College of Medicine, Seoul, South Korea; ^3^College of Pharmacy and Integrated Research Institute for Drug Development, Dongguk University, Seoul, South Korea

**Keywords:** ε-viniferin, antimicobacterial, antibiofilm, *Streptococcus pneumoniae*, cell membrane

## Abstract

Polyphenol ε-viniferin (**2**) is a protective phytochemical found in several plant families. Here, we report a simple and effective method for the synthesis of (±)-ε-viniferin (**2**) as major product and (±)-(E)-ω-viniferin (**3**) as a minor product. Synthesized viniferin compounds and standard viniferin were analyzed for antibacterial and antibiofilm activity against Gram-positive bacteria *Streptococcus pneumoniae*. The minimum inhibitory concentrations (MICs) of (±)-ε-viniferin (**2**) and standard viniferin were 20 µm. However, the MICs of (±)-(E)-ω-viniferin (**3**) and compound **8** were 40 µm. Although viniferin significantly (p < 0.05) reduced pre-established *in vitro* biofilms and killed bacteria within the biofilm, it was unable to prevent biofilm formation at sub-MIC concentrations. The time kill experiment revealed that viniferin killed bacteria and reduced 2.8 log_10_ bacteria at 2 × MIC concentration after 24 h. Scanning electron microscope (SEM) analysis and live/dead biofilm staining of pre-established biofilms revealed that viniferin treatment disrupts membrane integrity of biofilm bacteria. Crystal violet absorption, total protein, and DNA and RNA release revealed that viniferin alters bacterial cell permeability, eventually killing bacteria.

## Introduction

*Streptococcus pneumoniae* (*S. pneumoniae*) asymptotically colonizes the nosocomial cavity, causing infections under immune-compressed conditions. Pneumococci initially colonize nasopharyngeal mucosa in the form of biofilms (Bogaert et al., 2004; Simell et al., 2012). Biofilms in the nasopharyngeal cavity serve as reservoirs of bacteria that transit to sterile sites, causing infections such as otitis media (OM) pneumonia, bacteremia, meningitis, and sepsis (Wardlaw et al., 2006; Bergenfelz and Hakansson, 2017). Pneumococcal biofilms have been detected in infected sites such as tympanostomy tubes, human mucosa biopsies, and resected adenoids (Hall-Stoodley et al., 2006; Hoa et al., 2009). *S. pneumoniae* biofilms were also detected in the middle ear and nasopharynx of experimentally infected cinchona and mice (Reid et al., 2009; Sanchez et al., 2010). Bacteria in the biofilms were found in a self-produced matrix consisting of an extracellular polymeric substance (EPS) that confers resistance against conventional antibiotics and host immune defense (Donlan and Costerton, 2002). In mature biofilms, the EPS matrix resists antibiotic penetration via: i) charged polymers that prevent drug diffusion or ii) restriction of antimicrobial agents to the surface, leaving deeper cells unaffected, causing bacteria persistence (Marsh, 2004). Also, compared to planktonic bacteria, biofilm bacteria exhibit differential gene expression by adopting metabolic pathways less sensitive to the quinolone class of antibiotics and β-lactams, which target macromolecules or metabolic pathways (Xie et al., 2005; Mascio et al., 2007; Yadav et al., 2012). Antimicrobials inhibiting normal cells are made ineffective due to bacteria in biofilms growing slowly under nutrient- and aeration-depleted conditions (Davies, 2003). Resistance against commonly used antibiotics hampers treatment options (Korona-Glowniak et al., 2018). Consequently, new safe antimicrobials or antibiofilm agents that limit emergence of antibiotic-resistant bacteria are needed (Hurdle et al., 2011). Reportedly, novel phyto-compounds display potential to mitigate bacterial growth, including both pneumococcal planktonic and biofilm growth (Kwieciński et al., 2009; Ma et al., 2012; Quave et al., 2012; Burt et al., 2014; Yadav et al., 2015; Ma et al., 2018).

**Figure 1:**
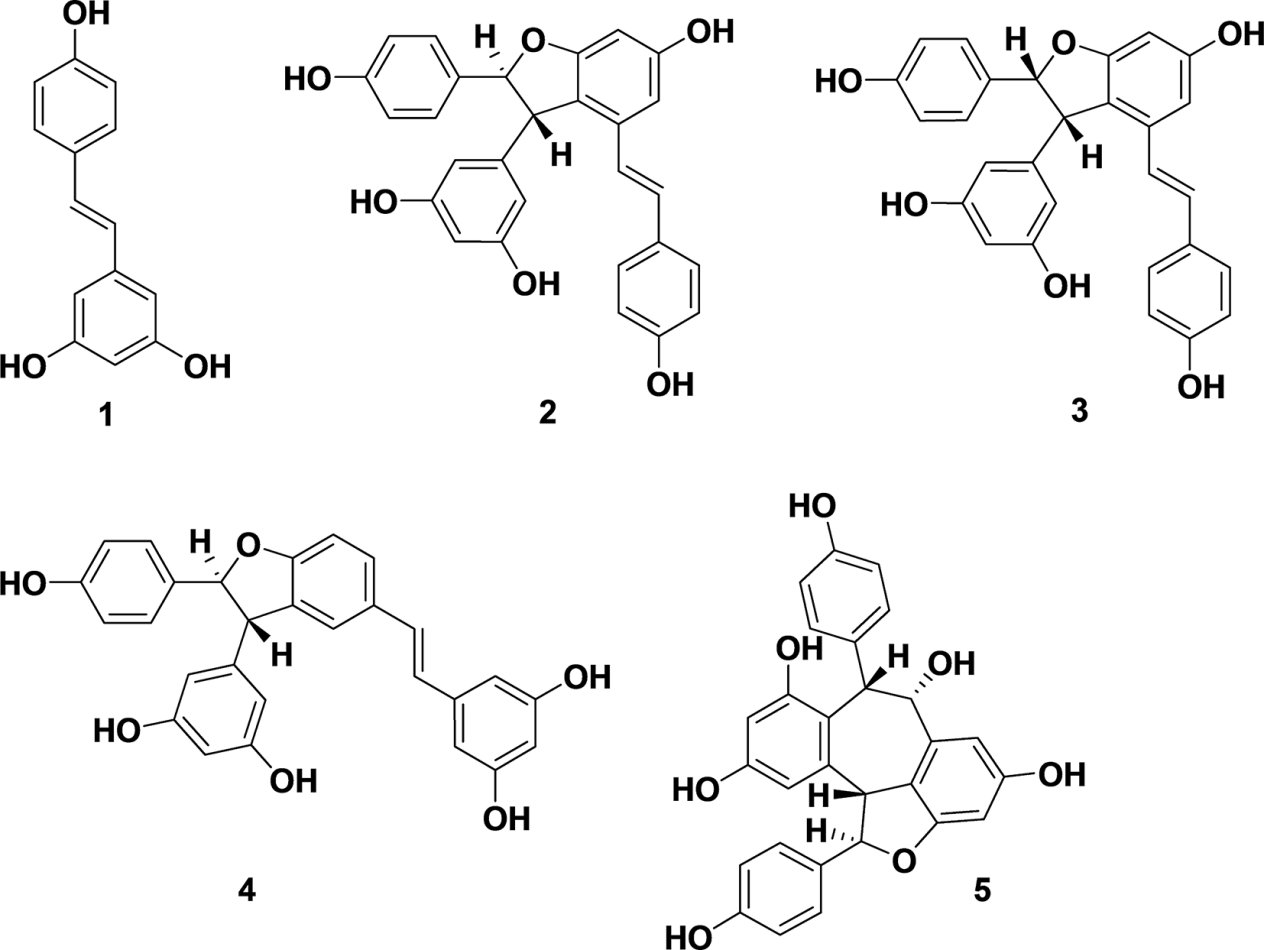
Biologically active stilbenes: resveratrol (**1**), ε-viniferin (**2**), (E)-ω-viniferin (**3**), δ-viniferin (**4**), and balanocarpol (**5**).

Natural polyphenols exhibit a range of biological activities (Lee et al., 2014). Stilbenes, known for antioxidant, anti-fungal and antimicrobial activities, are natural products used in chemotherapy (Shimizu et al., 2000). The antimicrobial activity and mechanism of stilbene monomer, *trans*-resveratrol (**1**), was investigated (Hwang and Lim, 2015). Oligostilbenes (**Figure 1**), produced *via* oxidative oligomerization of phenols, display various biological activities (Sotheeswaran and Pasupathy, 1993; Cichewicz and Kouzi, 2002). A dimeric product of *trans*-resveratrol (**1**), ε-viniferin (**2**), found in many plant families including *Vitaceae*, grapvines (*Vitis*), and *Carex*, exhibits P450 inhibitory antioxidants, as well as hepato-protective and antimicrobial activities (Fiorentino et al., 2008; Santamaria et al., 2012). Naturally occurring ε-viniferin, in *Carex pumila* and *C. lactiflora* extracts, displays antibiofilm activities against Gram-negative bacteria such as *Escherichia (E. coli)* (Lee et al., 2014). However, antimicrobial and antibiofilm activities of synthetic ε-viniferin and its derivatives against Gram-positive bacteria remain unknown.

Few reports are available on the synthesis of ε-viniferin. However, related natural products have been synthesized. Oxidative cyclization of resveratrol using FeCl_3_·6H_2_O and Ti (NO_3_)_3_ in methanol has been reported (Yao et al., 2004; Takaya et al., 2005). Although a method for total synthesis of ε-viniferin has been reported, it only yields low amounts of viniferin and requires more steps (Lindgen et al., 2016). To overcome these limitations, we synthesized ε-viniferin (**2**) and (*E*)-ω-viniferin (**3**) as racemates using ruthenium chloride–induced oxidative cyclization of trans-resveratrol. Compounds **8** and **9** were also synthesized from ε-viniferin (**2**). This novel method is economical and improves yields for large-scale synthesis. Synthetic viniferins were evaluated for antimicrobial and antibiofilm activity against *S. pneumoniae* planktonic and biofilm states.

## Materials and Methods

### Synthesis of Viniferins

All chemicals were purchased commercially and used without further purification. Flash column chromatography enabled product purification, and thin layer chromatography (TLC) analysis was conducted on commercial plates coated with silica gel 60 F_254_. Visualization of spots on TLC plates was achieved *via* ultraviolet (UV) radiation. Mass spectra were recorded using high-resolution mass spectrometry (HRMS, electrospray ionization MS) obtained on a G2 quadrupole time-of-flight (QTOF) mass spectrometer. Proton nuclear magnetic resonance (^1^H NMR) spectra were determined on a Varian (400 MHz) spectrometer (Varian Medical Systems, Inc., Palo Alto, CA, USA). ^13^C NMR spectra were recorded on a Varian (100 MHz) spectrometer using tetramethylsilane (TMS) as an internal standard. Chemical shifts are provided in parts per million (ppm) downfield with coupling constants in hertz (Hz). Standard abbreviations s, d, t, and m refer to singlet, doublet, triplet, and multiplet, respectively. Infrared (IR) spectra were recorded on FT-IR (NICOLET-iS5). Final product purity was checked by reversed-phase high-pressure liquid chromatography (RP-HPLC) performed on the YL9100 YoungLin HPLC system equipped with an UV detector set at 280 and 254 nm. Mobile phases used were: (A) H_2_O containing 0.1% trifluoroacetic acid and (B) acetonitrile containing 0.1% trifluoroacetic acid. HPLC chromatogram for purity check was: 5–55% B for 0–12 min, 55–100% B for 12–15 min, 100% B for 15–19 min, 100–5% B for 19–22 min, and 5% B for 22–23 min. HPLC employed an Agilent Eclipse Plus C18 reverse-phase column (3.5 μm particle size) that was 4.6 in diameter × 100 mm in size with a flow rate of 1.0 ml/min. Starter chemicals such as resveratrol and RuCl_3_.H_2_O were purchased from Sigma Aldrich, TCI, Alfa-Aesar, and Combi Blocks. The purity of all biologically evaluated compounds was >95% (per HPLC).

#### Experimental Procedure

##### 1. (±)-ε-Viniferin Penta-Acetate (6) and (±)-(E)-ω-Viniferin Penta-Acetate (7)

Ruthenium (III) chloride (1.09 g, 5.26 mmol) was added to a stirred solution of resveratrol (1.0 g, 4.38 mmol) in methanol/water (10:1, 11 ml) at 0°C. The reaction mass was stirred at 35°C for 3 h. After removing methanol in vacuum, the crude residue was dissolved in ethyl acetate (200 ml) and washed with sat. brine solution. The organic layer was dried over anhydrous MgSO_4_, filtered, and evaporated. The crude residue was purified *via* silica gel column chromatography (0–20% acetone in methylene chloride) to obtain an ε- and ω-viniferin–enriched product (340 mg) and unreacted resveratrol (350 mg, 1.53 mmol). The ε- and ω-viniferin–enriched products were dissolved in dichloromethane (20 ml) and dimethyl sulfoxide (DMSO) (1 ml). Triethylamine (TMS) (2.8 ml, 17.8 mmol) and Ac_2_O (1.36 ml, 14.4 mmol) were added at 0 °C and stirred at room temperature (RT) for 5 h. The reaction mixture was diluted with methylene chloride (100 ml) and washed using sat. NaHCO_3_. The organic layer was washed with sat. brine, dried over MgSO_4_, filtered, and evaporated. The crude product was purified *via* silica gel column chromatography (15–25% ethyl acetate in hexane) to afford ε-viniferin penta-acetate (**6**) (0.18 g, 18%) as a white solid and ω-viniferin penta-acetate (**7**) (75 mg, 7.5%) as a white solid following 2 steps.

Analytical data of ε-viniferin penta-acetate (**6**): ^1^H NMR (400 MHz, CDCl_3_) δ (ppm) 7.3 (d, *J* = 8.8 Hz, 2 H), 7.18 (d, *J* = 8.4 Hz, 2 H), 7.1 (d, *J* = 8.4 Hz, 2 H), 6.99 (d, *J* = 8.4 Hz, 2 H), 6.94 (s, 1 H), 6.9 (s, 1 H), 6.89 (d, *J* = 16.8 Hz, 1 H), 6.85 (s, 2 H), 6.64 (s, 1 H), 6.55 (d, *J* = 16 Hz, 1 H), 5.6 (d, *J* = 6.8 Hz, 1 H), 4.6 (d, *J* = 6.4 Hz, 1 H), 2.33 (s, 3 H), 2.29 (s, 3 H), 2.26 (bs, 9 H).

Analytical data of ω-viniferin penta-acetate (**7**): ^1^H NMR (400 MHz, CDCl_3_) δ (ppm) 7.27 (d, *J* = 8.4 Hz, 2 H), 7.12 (d, *J* = 8.4 Hz, 2 H), 7.0 (d, *J* = 8.4 Hz, 2 H), 6.96 (d, *J* = 2.0 Hz, 1 H), 6.94 (d, *J* = 15.6 Hz, 1 H), 6.92 (d, *J* = 9.2 Hz, 2 H), 6.7 (d, *J* = 2.0 Hz, 1 H), 6.68 (d, *J* = 16.4 Hz, 1 H), 6.63 (t, *J* = 1.8 Hz, 1 H), 6.37 (d, *J* = 1.6 Hz, 2 H), 6.0 (d, *J* = 8.8 Hz, 1 H), 4.82 (d, *J* = 8.8 Hz, 1 H), 2.35 (s, 3 H), 2.27 (s, 3 H), 2.23 (s, 3 H), 2.18 (s, 6 H).

##### 2. (±)-ε-Viniferin (2)

KOH (25 mg, 0.44 mmol) was added to a stirred solution of **6** (50 mg, 0.075 mmol) in methanol (6 ml), at RT. Reaction mass was stirred for 30 min. After removing methanol in a vacuum, crude residue was dissolved in ethyl acetate (25 ml) and 1N HCl (10 ml). The organic layer was dried over anhydrous MgSO_4_, filtered, and evaporated. Crude residue was purified *via* recrystallization (acetone: methylene chloride), producing (±)-ε-viniferin (**2**) (30 mg, 88%) as a pale yellow-colored solid. ^1^H NMR (400 MHz, CD_3_OD) δ (ppm) 7.14 (d, *J* = 8.8 Hz, 2 H), 7.04 (d, *J* = 8 Hz, 2 H), 6.83 (d, *J* = 16.4 Hz, 1 H), 6.75 (d, *J* = 8.4 Hz, 2 H), 6.65 (d, *J* = 8.4 Hz, 2 H), 6.62 (d, *J* = 1.6 Hz, 1 H), 6.58 (d, *J* = 16.4 Hz, 1 H), 6.24 (d, *J* = 1.6 Hz, 1 H), 6.17 (d, *J* = 2 Hz, 1 H), 6.16 (bs, 2 H), 5.37 (d, *J* = 6.8 Hz, 1 H), 4.35 (d, *J* = 6.8 Hz, 1 H); ^13^C NMR (100 MHz, CD_3_OD) δ (ppm) 161.3, 158.3–156.9, 145.9, 135.5, 132.4, 128.9, 127.3, 126.7, 122.2, 118.6, 114.9, 106, 102.9, 100.7, 95.4, 93.4, 56.8; IR (cm^-1^): 3311, 1590, 1440, 1237, 1148, 998, 824 HRMS (ESI) *m/z* calcd for C_28_H_23_O_6_ [M+H]+: 455.1495, found: 455.1487; purity 95.4% (as determined by RP-HPLC, *t*_R_ = 11.05 min).

##### 3. (±)-E-ω-Viniferin (3)

Compound **3** was obtained as a brown-colored solid (18 mg, 75%) from compound **7** (30 mg), according to the procedure for compound **2**. ^1^H NMR (400 MHz, CD_3_OD) δ (ppm) 7.13 (d, *J* = 8.4 Hz, 2 H), 6.97 (d, *J* = 8.8 Hz, 2 H), 6.88 (d, *J* = 16.4 Hz, 1 H), 6.67 (d, *J* = 8.4 Hz, 2 H), 6.67 (d, *J* = 16.4 Hz, 1 H), 6.63 (d, *J* = 2.0 Hz, 1 H), 6.56 (d, *J* = 8.8 Hz, 2 H), 6.3 (d, *J* = 1.2 Hz, 1 H), 5.92 (t, *J* = 2.4 Hz, 1 H), 5.84 (d, *J* = 8.4 Hz, 1 H), 5.75 (bs, 2 H), 4.6 (d, *J* = 8.4 Hz, 1 H); ^13^C NMR (100 MHz, CD_3_OD) δ (ppm) 161.2, 157.9, 157.3, 156.9, 156, 142.3, 135.4, 129.4, 128.9, 128.2, 127.8, 127.3, 122.5, 120.9, 115, 113.8, 107.7, 103.7, 100.2, 95.7, 89.4, 52.1; IR (cm^-1^): 3311, 1621, 1512, 1442, 1226, 1120, 992; HRMS (ESI) m/z calcd for C_28_H_23_O_6_ [M+H]+: 455.1495, found: 455.1495; purity 96.2% (as determined by RP-HPLC, *t*_R_ = 11.4 min).

##### 4. Compound (8)

Pd/C (10%, 4.4 mg) was added to a stirred solution of **2** (22 mg, 0.048 mmol) in ethanol (1 ml), in a flask sealed with a hydrogen balloon. The suspension was stirred for 3 h at RT. The resulting suspension was filtered through a pad of celite, and the filtrate was evaporated. The crude residue was purified *via* silica gel column chromatogra (ppm) phy (0–5% MeOH in methylene chloride) to afford **8** (8.4 mg, 38%) as a brown-colored solid. ^1^H NMR (400 MHz, CD_3_OD) δ (ppm) 7.03 (d, *J* = 8.4 Hz, 2 H), 6.74 (d, *J* = 8.4 Hz, 2 H), 6.69 (d, *J* = 8.4 Hz, 2 H), 6.57 (d, *J* = 8 Hz, 2 H), 6.17 (s, 1 H), 6.15 (bs, 2 H), 6.06 (bs, 2 H), 5.23 (d, *J* = 6.4 Hz, 1 H), 3.95 (d, *J* = 6.4 Hz, 1 H), 2.47–2.38 (bm, 4 H); ^13^C NMR (100 MHz, CD_3_OD) δ (ppm) 160.8, 158.5, 158.1, 157, 154.9, 146.1, 139.9, 132.4, 129, 126.9, 119.2, 114.8, 114.4, 108.1, 106, 100.6, 94.1, 93.4, 56.5, 35.6, 35; IR (cm^-1^): 3261, 1616, 1440, 1224, 1117, 1000; HRMS (ESI) *m/z* calcd for C_28_H_25_O_6_ [M+H]+: 457.1651, found: 457.1758; purity 100% (as determined by RP-HPLC, *t*_R_ = 10.78 min).

##### 5. Compound (9)

K_2_CO_3_ (92 mg, 0.06 mmol) and MeI (0.31 g, 2.2 mmol) were added to a stirred solution of compound **2** (100 mg, 0.22 mmol) in acetone (5 ml). The reaction suspension was slowly heated to 65°C and maintained overnight. The resulting suspension was filtered through a celite pad and the filtrate evaporated. The residue was dissolved in ethyl acetate (50 ml), washed with water, sat. brine, dried over MgSO_4_, filtered, and evaporated. The crude product was purified by silica gel column chromatography (hexane/ethyl acetate = 8:2, v/v) to afford **9** (20 mg, 17%) as an off-white solid. ^1^H NMR (400 MHz, CDCl_3_) δ (ppm) 7.27 (d, *J* = 8.4 Hz, 2 H), 7.13 (d, *J* = 8.8 Hz, 2 H), 6.88 (d, *J* = 16.8 Hz, 1 H), 6.86 (d, *J* = 8.8 Hz, 2 H), 6.79 (d, *J* = 8.8 Hz, 2 H), 6.72 (d, *J* = 2 Hz, 1 H), 6.61 (d, *J* = 16.8 Hz, 1 H), 6.46 (d, *J* = 2.4 Hz, 1 H), 6.36 (bs, 3 H), 5.52 (d, *J* = 5.6 Hz, 1 H), 4.51 (d, *J* = 5.6 Hz, 1 H), 3.86 (s, 3 H), 3.79 (s, 3 H), 3.77 (s, 3 H), 3.73 (bs, 6 H); ^13^C NMR (100 MHz, CDCl_3_) δ (ppm) 161.3–161.1, 159.4, 159.2, 145.6, 135.3, 133.6, 129.8, 129.3, 127.6, 126.9, 123.1, 119.5, 114, 105.8, 102.3, 98.8, 95, 93, 56.9, 55.5–55.2; HRMS (ESI) m/z calcd. for C_33_H_33_O_6_ [M+H]+: 525.2277, found: 525.2383; purity 100% (as determined by RP-HPLC, *t*_R_ = 17.7 min).

### Bacteria Strain and Culture Medium

*S. pneumonia* [National Collection of Type Cultures (NCTC) 7466] was purchased from the Health Protection Agency Culture Collection (Salisbury, UK). *S. pneumoniae* National Collection of Type Cultures (NCTC) 7466 is Avery’s virulent strain D39, serotype 2, encapsulated strain (Avery et al., 1944). *S. pneumoniae* CCARM 4003, an antibiotic resistance strain (clindamycin >128, erythromycin >512, tetracycline 16 µg/mL), was purchased from the Culture Collection of Antimicrobial Resistance Microorganisms (CCARM 4003, Seoul, South Korea). *S. pneumoniae* strain 11 (strain 7101975) was obtained from the infectious disease center, Korea University College of Medicine, Seoul; serotype 3 (ATCC 6303), 19F (ATCC 49619) and 19A, and R6 were purchased from the American Type Culture Collection (Manassas, VA, USA) (Yadav et al., 2017). Bacteria were grown on an agar plate supplemented with 5% defibrinated sheep blood agar plates (BAPs) purchased from Shin Yang Chemicals Co., Ltd., Seoul, Korea and in brain heart infusion (BHI) broth.

### Minimum Inhibitory Concentration (MIC) Detection

MICs were determined using the broth microdilution method recommended by the Clinical and Laboratory Standards Institute (CLSI, 2005). *S. pneumoniae* D39 colonies grown on BAP were scraped and grown in MH broth until log phase. Log-phase cells were diluted to prepare bacterial suspensions containing 1–5 × 10^5^ colony-forming units (cfu)/ml. Viniferin solutions were added to Mueller Hinton Broth (MHB) cell suspension from 10 to 50 µm concentrations. Next, 200 µl of cell suspension along with viniferin solutions were inoculated in each well of a 96-well plate. Negative control samples were supplied with DMSO. Plates were incubated for 18 h at 37°C. Bacterial growth was detected by measuring optical density at 600 nm using a microplate reader. MIC was defined as the lowest concentration of viniferin solution at which no visible bacterial growth was observed. Alternately, MICs were confirmed by determining bacterial cfu counts. Cell suspensions were diluted two-fold, plated on BAP agar plates, and incubated overnight at 37°C, and cfu were counted. The experiment was repeated twice in triplicate. The inhibitory effect of 20 µm viniferin on strains resistant to erythromycin (> 512 µg/ml) and different serotypes (2, 3, 19A, 19F) of *S. pneumoniae* was evaluated.

### Effect of Viniferin on Biofilm Inhibition

The effect of ε-viniferin (**2**) on *S. pneumoniae* biofilm growth was evaluated *in vitro* using static biofilm models, and biofilm biomass was quantified using crystal violet (CV) microtiter plate assays (Christensen et al., 1985; Yadav et al., 2017). Bacteria grown on blood agar plates were scraped and grown to log phase in BHI broth. Freshly prepared bacterial cell suspensions were diluted 1:200 and inoculated at 200 µl in 96-well plates or at 1 ml in 24-well plates. Viniferin solution was added to each well at concentrations from 10 to 50 µm. The control samples (vehicle) were treated with similar volumes of DMSO (0.1 to 0.5%). Plates were incubated at 37°C for 18 h. Following incubation, the medium and planktonic cells were decanted and the plate washed twice with phosphate-buffered saline (PBS). Biofilms at the bottom of the plates were stained using 50 µl of 0.1% CV for 15 min. The biofilms were washed twice with PBS and air-dried, and CV was dissolved in ethanol (200 µl ethanol for 96-well or 1 ml for 24-well plate). Absorbance of the dissolved CV was measured at 570 nm using microplate readers. To evaluate the effects of viniferin on planktonic growth, *S. pneumoniae* D39 were grown in different concentrations of synthetic ε-viniferin (**2**) (MIC, 0.5 × MIC, and 0.25 × MIC). Growth was detected by measuring absorbance at 600 nm at different time intervals.

### Effect of Viniferin on Eradication of Pre-Established Biofilms

Microbial biofilms are difficult to eradicate, and several-fold-high concentrations of antibiotics are needed to eradicate pre-established biofilms (Høiby et al., 2010; Co et al., 2018). We evaluated *in vitro* biofilm eradication potential of viniferin. *In vitro* biofilms of *S. pneumoniae* were grown in 24-well plates for 18 h *via* procedures described above. These biofilms were treated with MIC and 2 × MIC concentrations of viniferin for 6 h. Biofilm biomasses were quantified using CV microtiter plate assays, and viable bacteria were detected by cfu counts as described above. Daptomycin, a lipopeptide antibiotic known to eradicate biofilm, was used as a positive control.

Metabolically active bacteria within pre-established biofilms were detected using resazurin staining. Resazurin is a blue-colored, non-fluorescent dye, and in the presence of the metabolically active cells, it gets reduced to a pink and highly fluorescent compound called resorufin. The fluorescent detection allows the quantitative measurement of cell viability. Resazurin staining was performed as per previously reported procedure with minor modification (Paytubi et al., 2017). 0.02% (w/v) resazurin sodium salt (Sigma, USA) solution was prepared in sterile distilled water and filter-sterilized. Pre-established biofilms grown in 24-well plates and treated with viniferin as mentioned above were dissolved in sterile water, and 100 μl was transferred to a 96-well plate. The biofilm suspensions were incubated with 25 μl (0.02%) resazurin dye and 100 μl BHI broth. The plate was incubated at 37°C for 2 h, and fluorescence was measured (excitation 530 nm, emission 590 nm) using a microplate reader (Thermo Scientific, Waltham, MA, USA).

### SEM Analysis of Pre-Established Biofilms

*S. pneumoniae* form robust biofilms of significant depth *in vitro* (Moscoso et al., 2006). We evaluated changes in *S. pneumoniae in vitro* biofilm morphology upon viniferin treatment using SEM. Synthetic ε-viniferin (**2**) was the most active; therefore, experiments were performed with ε-viniferin (**2**). *In vitro* biofilms were grown in 24-well plates for 18 h *via* procedures described above and treated with 2 × MIC concentration of ε-viniferin (**2**) for 6 h. Control biofilms were treated with DMSO. Biofilms were washed with PBS pre-fixed by 2% glutaraldehyde + formaldehyde solution and post fixed with osmic acid (2%) for 2 h. The biofilms were dehydrated *via* increasing ethanol concentration gradients (60–95%) followed by *t*-butyl alcohol treatment. The samples were freeze-dried and coated with platinum. Images were captured using field emission SEM (FE-SEM; Hitachi, S-4700, Tokyo, Japan).

### Confocal Microscopy Analysis of Pre-Established Biofilms

Alteration of pre-established biofilm structure and viability upon viniferin treatment was analyzed using live/dead biofilm staining and confocal microscopy. *S. pneumoniae* D39 biofilms were grown on micro-discs for 18 h *via* procedures described above, and treated with ε-viniferin (**2**) (2 × MIC) for 6 h. The biofilms were stained with a LIVE/DEAD biofilm viability kit (Invitrogen, California, USA) according to the manufacturer’s instructions. Control biofilms were treated with similar volumes of DMSO. After washing, stained biofilms were viewed under a Nikon A1 confocal microscope (Nikon Instruments Inc., NY, USA) using fluorescein (green) and Texas red (red) band-pass filter sets.

### Effect of Viniferin on Bacteria Killing

To evaluate the bactericidal or bacteriostatic effect of viniferin on *S. pneumoniae*, a bacteria-killing experiment was performed. An *S. pneumoniae* D39 cell suspension was grown in BHI broth up to early log phase, and aliquots were treated with 2 × MIC concentrations of viniferin (compounds **2**, **3**, and **8**, and ε-viniferin standard). Cells were further grown at 37°C, and viable bacteria were detected at different time points (0, 6, 12, and 24 h) *via* cfu counts as described above. Control samples were treated with DMSO.

### Crystal Violet Absorption Assay

Alteration of bacterial membrane permeability upon viniferin treatment was evaluated using CV absorption assays (Devi et al., 2010). *S. pneumoniae* D39 colonies were grown in BHI broth up to early log phase. The cells were pelleted *via* centrifugation at 4,500*g* for 5 min at 4°C. Bacterial cells were washed with PBS and treated with ε-viniferin (**2**) and erythromycin (MIC and 2 × MIC) for 3 h at 37°C. Erythromycin was used as the control, as it had no effect on bacterial membrane permeability (non-dividing cells). Negative control samples were treated with DMSO. Following incubation, cells were harvested *via* centrifugation at 9300*g* for 5 min and suspended in 10 µl/ml CV solution (0.01%). The cell suspension was incubated for 10 min and centrifuged at 13,400*g* for 15 min. The OD of the supernatant was detected at 590 nm using a microplate reader. The OD value of the original solution was considered 100%, and the percentage of CV absorption was detected as follows:

$$\frac{\text{OD value of the sample}}{\text{OD value of the crystal violet solution}}\times 100$$

### Leakage of Total Proteins Through the Bacterial Membrane

Integrity of *S. pneumoniae* cell membrane upon viniferin treatment was examined by determining total protein release in supernatants. Protein concentrations in the supernatants were evaluated using the Pierce bicinchoninic acid (BCA) protein assay kit (Thermo Scientific, MA, USA). *S. pneumoniae* were grown to log phase, pelleted *via* centrifugation, and suspended in PBS. Cell suspensions (approx. 10^7^/ml) were treated with viniferin (MIC and 2 × MIC) and incubated at 37°C for 3 h. Next, cells were pelleted, and supernatant was filtered through a 0.2 µm syringe filter. The cell-free supernatants were treated with 200 µl each of solutions A and B (provided in kit) for 30 min, and absorbance at 562 nm was measured.

### Leakage of DNA and RNA Through the Bacterial Membrane

The integrity of the pneumococci’s cell membrane upon viniferin treatment was evaluated by monitoring release of cytoplasmic constituents such as DNA and RNA as per a previous report (Bouyahya et al., 2019). *S. pneumoniae* were grown up to mid-log phase in BHI medium. The cell suspensions were pelleted by centrifugation and dissolved in PBS. The cells were treated with viniferin (MIC and 2 × MIC) for 3 h at 37°C. After treatment, cells were pelleted by centrifugation (10,000*g* for 5 min), and supernatants were filter-sterilized using a 0.2 µm syringe filter. The released DNA and RNA in supernatants were determined by measuring optical density at 260 nm.

### Statistical Analysis

Experiments were performed in replicate, and means were calculated and compared with those of the control group. Statistical significance was assessed using Student’s *t*-tests, and a P < 0.05 was considered significant.

## Results

### Synthesis of (±)-ε-Viniferin (2), (±)-(E)-ω-Viniferin (3), 8, and 9

In the current study, resveratrol (**1**) was used as a precursor for the transformation with oxidizing agents. Initially, synthesis of ε-viniferin by oxidative coupling of resveratrol with known procedures was unsuccessful. The usefulness of ruthenium (III) chloride hydrate in the formation of the 2,3-dihydrobenzofuran unit is unknown, but the reagent is widely used to catalyze oxidation (like sulfite to sulfate) (Gao and Sharpless, 1988). Resveratrol (**1**) was treated with RuCl_3_·H_2_O in MeOH/water (10:1) at 0°C to 35°C to yield crude (±)-ε-viniferin (**2**) as a major product and (±)-(E)-ω-viniferin (**3**) as a minor product along with unconverted **1**. Simple column chromatography provided viniferin-enriched fractions. For purification purposes, crude column fractions were acetylated in methylene chloride (MC) to provide pure penta-acetate viniferins (**6** and **7**), and subsequent hydrolysis yielded **2** and **3**, respectively. Compounds **8** and **9** were also synthesized *via* compound **2**. Hydrogenation of (±)-ε-viniferin (**2**) with of Pd/C (10%) in EtOH produced **8**, while penta-methylation of **2** with MeI/K_2_CO_3_ yielded compound **9**. Analytical data of (±)-ε-viniferin (**2**) and (±)-(E)-ω-viniferin (**3**) (^1^H NMR and ^13^C NMR, HRMS) were in agreement with standard products (Ito et al., 1999; Mattivi et al., 2011).

**Scheme 1:**
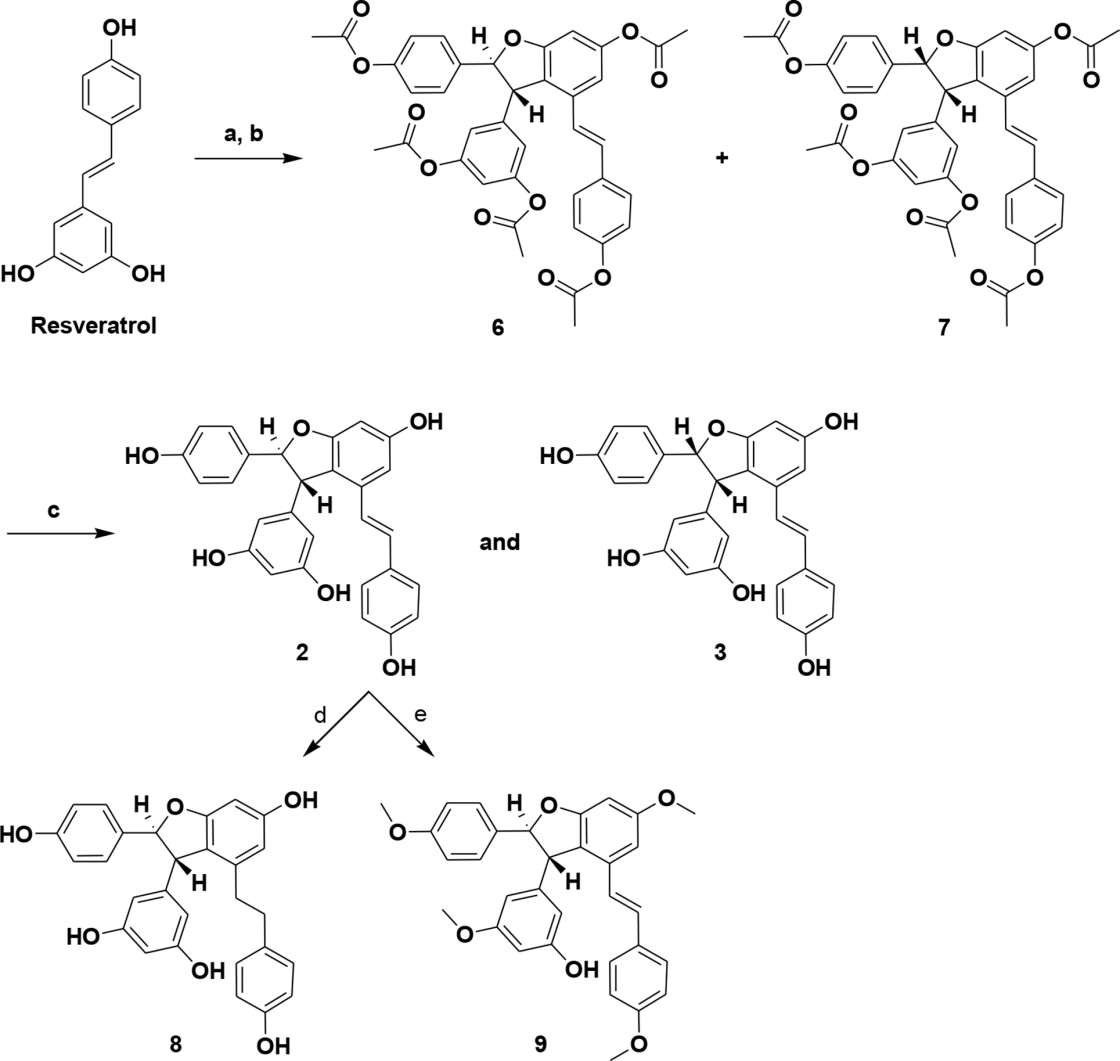
Synthesis of (±)-ε-viniferin (2), (±)-(E)-ω-viniferin (3), 8, and 9. **(a)** RuCl3·H2O, MeOH, water, 0°C to 35°C, 3 h; **(b)** (Ac)2O, TEA, MC, DMSO, 0°C to RT, 5 h; **(c)** KOH, MeOH, RT; **(d)** 10% Pd/C, H2, EtOH, RT, 3 h; **(e)** MeI, K2CO3, acetone, 65°C, 6 h.

### Minimum Inhibitory Concentration of Viniferin

**Figure 2:**
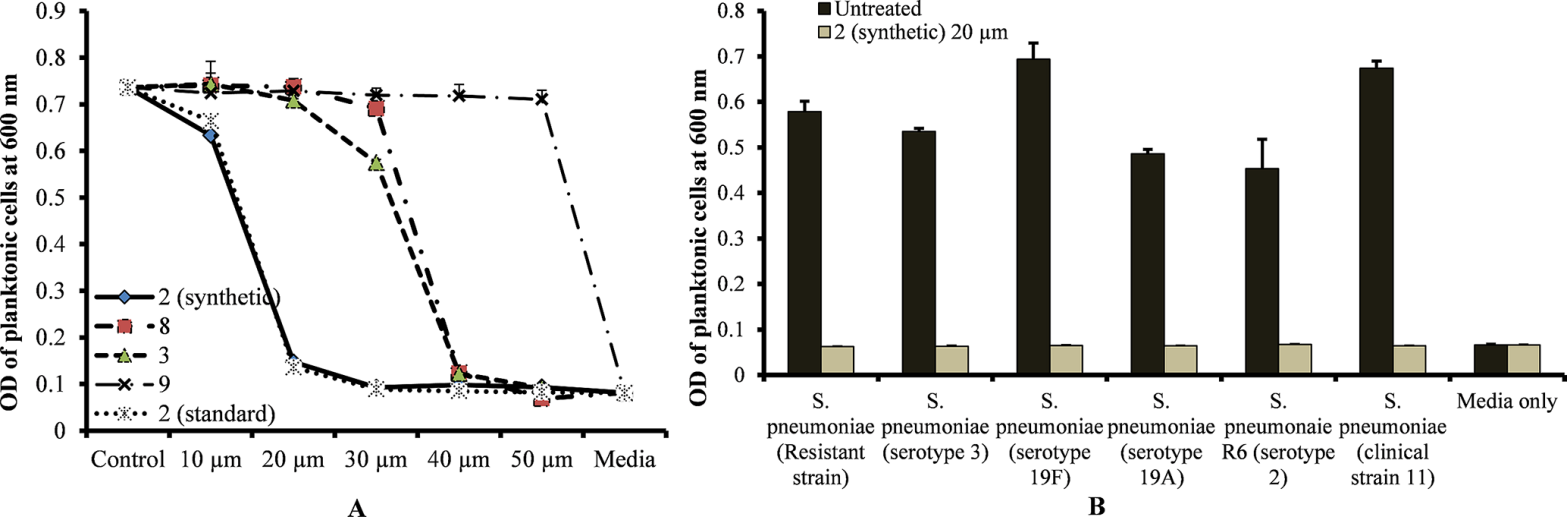
Detection of minimum inhibitory concentrations (MICs) of viniferin compounds against *S. pneumoniae*. **(A)** MIC detection of standard and synthetic viniferin compounds (**2**, **3**, **8**, and **9**) tested from 10 to 50 µm against *S. pneumoniae* D39 (serotype 2). **(B)** Inhibitory effect of synthetic ε-viniferin (**2**) (20 µm) on *S. pneumoniae* antibiotic resistance strain and different serotypes. Bacterial growth in the presence of viniferin was measured *via* optical density at 600 nm after 18 h. The means were calculated. Error bars represents standard deviations.

The MIC of standard viniferin and synthetic (±)-ε-viniferin (**2**) for *S. pneumoniae* was 20 µm, while the MIC of (±)-E-ω-viniferin (**3**) and **8** was 40 µm. However, concentrations of penta-methylated viniferin (**9**) up to 50 µm were unable to inhibit *S. pneumoniae* growth (**Figure 2A**). The most active compound, (±)-ε-viniferin, was tested against different *S. pneumoniae* serotypes. Results demonstrated that (±)-ε-viniferin inhibited the growth of *S. pneumonia* serotypes 2, 3, 19A, and 19F and clinical strains at 20 µm (**Figure 2B**). Similarly, the erythromycin-resistant strain of *S. pneumoniae* and R6 (un-encapsulated) were also inhibited by 20 µm synthetic (±)-ε-viniferin (**Figure 2B**).

### Viniferin Is Not Effective on *S. pneumoniae in Vitro* Biofilms at Sub-MIC

**Figure 3:**
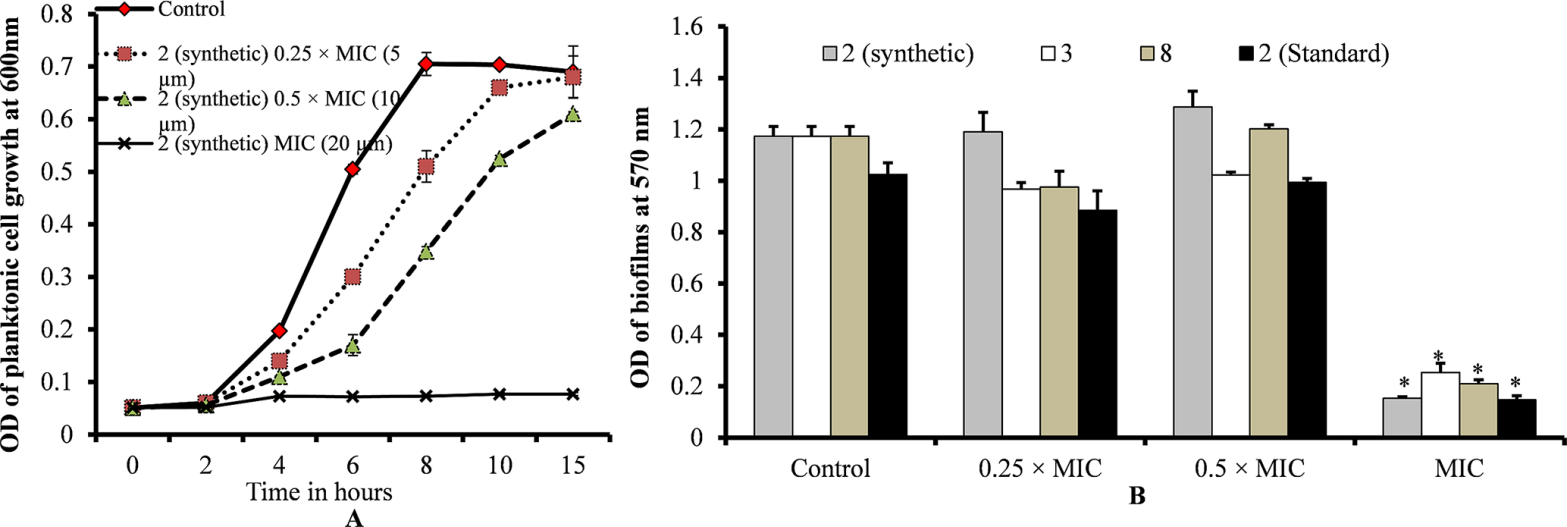
*S. pneumoniae* planktonic and *in vitro* biofilm growth in the presence of 0.25 × MIC or 0.5 × MIC or MIC of viniferins. **(A)** Planktonic growth was detected by measuring observance at 600 nm. **(B)** Biofilm biomass was detected *via* CV microplate assay. The error bars represent standard deviations. Statistical significance was calculated *via* Student’s *t*-test, and p < 0.05 was considered significant.

Planktonic growth results showed no bacterial growth at MIC and slow bacterial growth at 0.25 × MIC (sub-sub-MIC) or 0.5 × MIC (sub-MIC) of ε-viniferin (**2**) (**Figure 3A**). No significant difference was detected in the planktonic growth of bacteria between ε-viniferin (**2**) treated with 0.25 × MIC or 0.5 × MIC and the control following 15 h incubation (**Figure 3A**). Growing *S. pneumoniae in vitro* biofilms in presence of 0.25 × MIC or 0.5 × MIC of viniferin did not induce significant inhibition of biofilm growth (**Figure 3B**). However, at the MIC of viniferins, no biofilm growth was detected, which may have been due to complete inhibition of planktonic cell growth, which prevented the cells from reaching the number required for biofilm buildup. These results suggest that viniferin was unable to impede *in vitro* biofilms of *S. pneumoniae* at sub-MIC but hindered bacterial growth in planktonic form and biofilms at MIC.

### Viniferin Eradicated Pre-Established Biofilms

**Figure 4:**
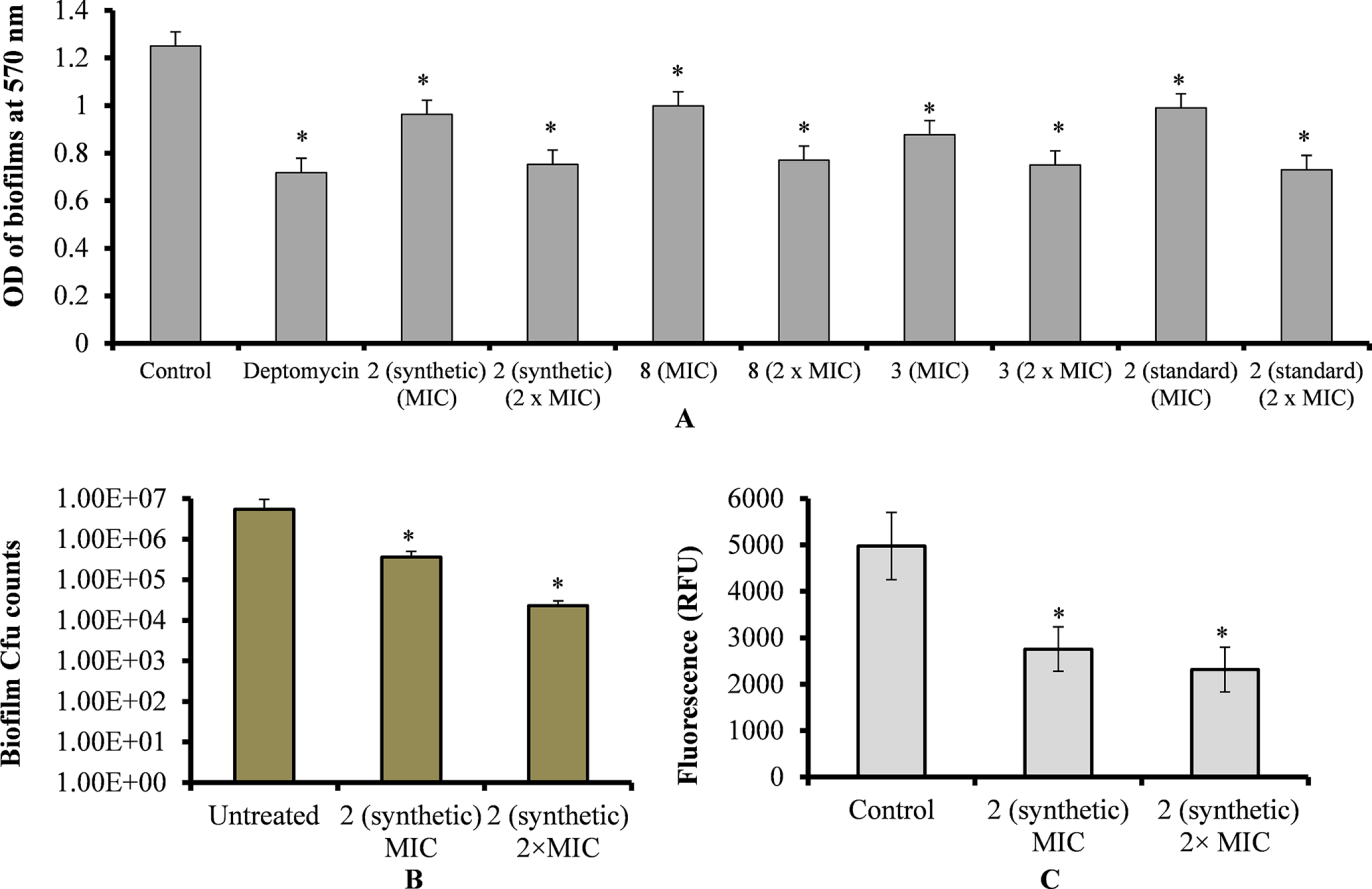
Effect of viniferins on pre-established biofilms of *S. pneumoniae*. **(A)** Treatment of pre-established biofilm with MIC and 2 × MIC of viniferins and biofilm biomass was quantified *via* CV microplate assay. **(B)** Treatment of pre-established biofilm with MIC and 2 × MIC of ε-viniferin (**2**) and viable bacteria detected by cfu counts. **(C)** Metabolically active bacteria within biofilms treated with MIC and 2 × MIC of ε-viniferin (**2**) *via* resazurin staining. The error bars represent standard deviations. Statistical significance was calculated by Student’s *t*-test, and * = *p* < 0.05 was considered significant.

Treatment of pre-established biofilms with viniferin compounds resulted in a significantly (p < 0.05) reduced biofilm biomass. At MIC and 2 × MIC, synthetic compounds **2**, **3**, and **8** and standard viniferin actively eradicated pre-established biofilms (**Figure 4A**). Treating biofilms with daptomycin (positive control) also eradicated >50% biofilms. Treatment of pre-established biofilms with MIC and 2 × MIC of ε-viniferin (**2**) significantly (p < 0.05) reduced viable bacteria in biofilm (**Figure 4B**). The resazurin staining results showed that the ε-viniferin (**2**) treatment (MIC and 2 × MIC) significantly (p < 0.05) reduced the metabolically active bacteria within pre-established biofilms (**Figure 4C**). These results indicated that viniferins effectively decrease pre-established biofilms of pneumococci and that ε-viniferin (**2**) was able to kill pneumococci in biofilms.

### Viniferin Treatment Distorts *S. pneumoniae* Within Biofilms

**Figure 5:**
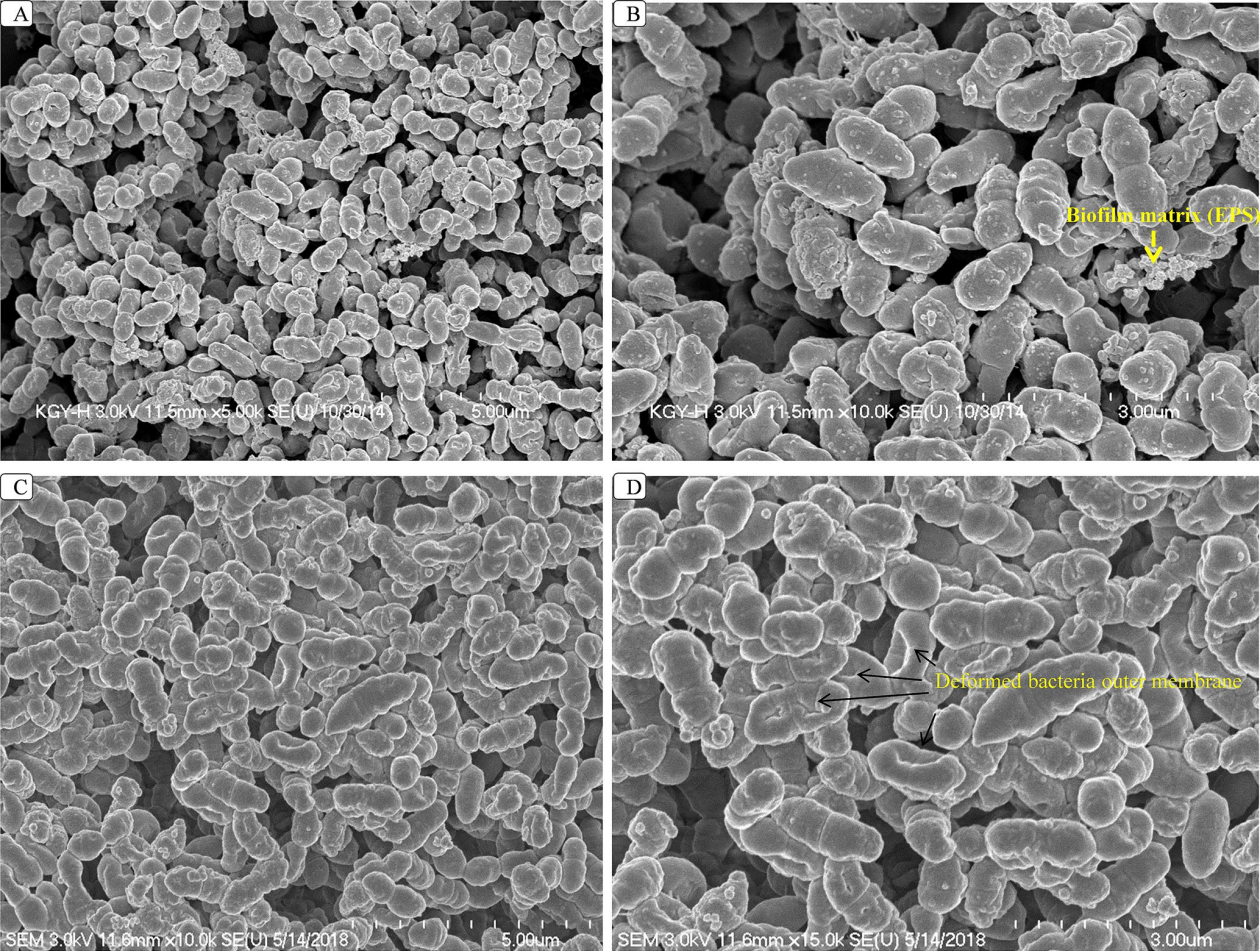
SEM analysis of *S. pneumoniae* biofilms treated with 2 × MIC of ε-viniferin (**2**). Images **A** and **B** are SEM images of control biofilms. Images **C** and **D** are SEM images of viniferin-treated biofilms. In viniferin-treated biofilms, cells appear shrunken, and cell membranes were damaged (indicated by arrow). SEM images were 5 and 3 µm.

Changes that occur in biofilm structures following viniferin treatment were evaluated using SEM. SEM revealed that intact three-dimensional biofilms with typical EPS matrices were deposited on the surface of bacteria in control samples (**Figures 5A, B**). However, the analysis also revealed that the structures of biofilms treated with ε-viniferin (**2**) were altered (**Figures 5C, D**). Cells in viniferin-treated biofilms were distorted, and the cell membrane appeared shrunken. The lysed cells (**Figure 5D**, arrow) indicated that viniferin effectively killed bacteria within the biofilm but was unable to completely disassemble the biofilm matrix. Also, the EPS present on bacterial surfaces in biofilms of control samples (**Figure 5B**, arrow) was absent on viniferin-treated biofilm bacterial surfaces.

### Confocal Microscopy Analysis of Pre-Established Biofilms

**Figure 6:**
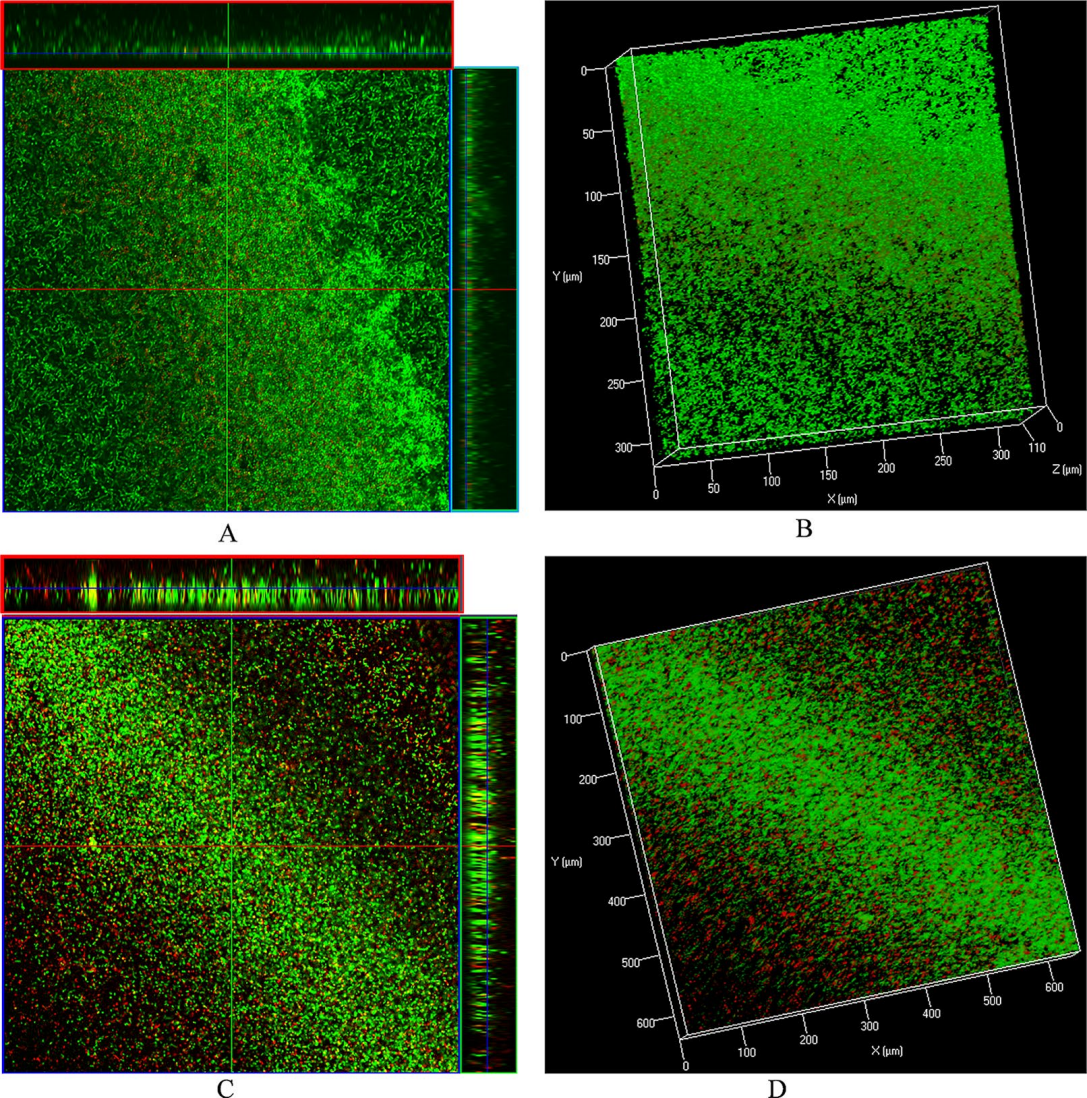
Live/dead biofilm viability staining and confocal microscopy of pre-established biofilms treated with ε-viniferin (**2**). **(A)** 2-dimensional (with ortho-section) confocal microscopy image of control biofilms and **(B)** 3-dimensional image of control biofilms. **(C)** 2-D (with ortho-section) confocal microscopy image of pre-established biofilm treated with 2 × MIC viniferin and **(D)** 3-D confocal microscopy image of viniferin-treated biofilms.

The LIVE/DEAD biofilm viability kit utilizes a mixture of the SYTO^®^ 9 green-fluorescent nucleic acid stain and the red-fluorescent nucleic acid stain, propidium iodide. Bacteria with intact cell membranes (live cells) are stained green, and those with damaged membranes are stained red. Live/dead biofilm staining revealed that control (DMSO) biofilms were compact with no visible dead cells (**Figures 6A, B**). However, biofilms treated with ε-viniferin (**2**) were thin, and dead cells (red) or cells with compromised membranes (yellow) were visible (**Figures 6C, D**), indicating that viniferin treatment kills pneumococci in biofilm.

### Viniferin Kills 99% of *Streptococcus pneumoniae* Bacteria

**Figure 7:**
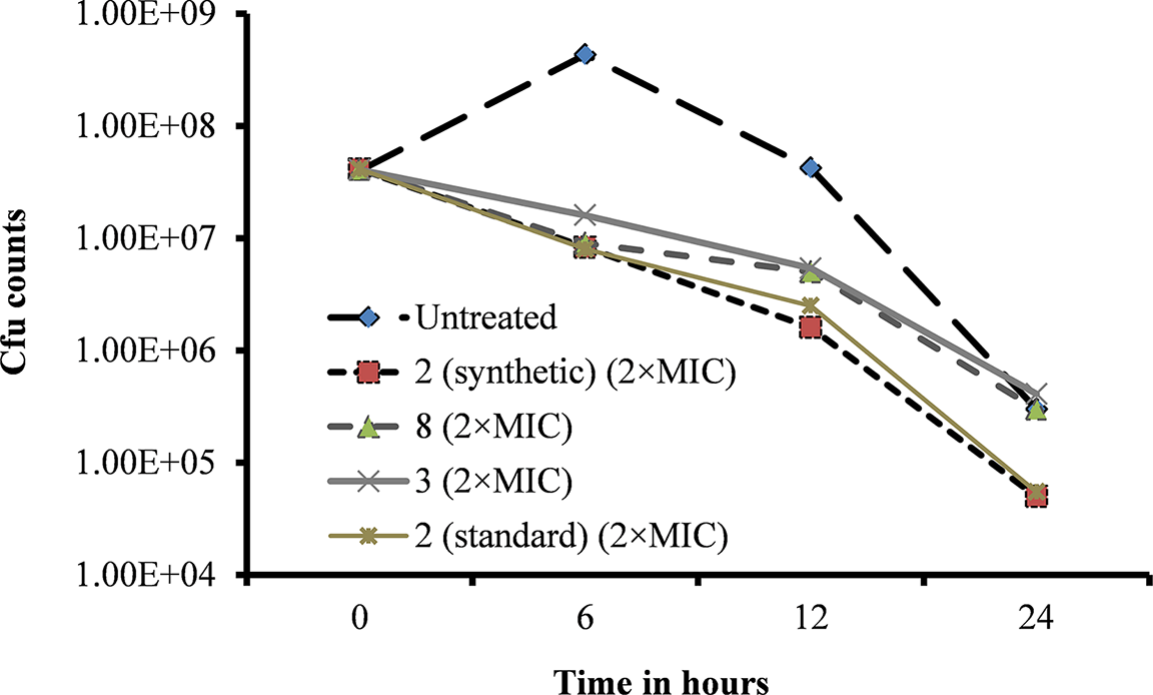
Effect of viniferins on viability of *S. pneumoniae* D39. Early-log-phase bacteria were treated with 2 × MIC concentration of viniferins, and bacterial viability was detected *via* cfu counts at 0, 6, 12, and 24 h.

A bacteria killing time experiment was performed to determine whether the inhibitory activity of viniferin is bacteriostatic or bactericidal. Untreated (control) bacterial growth was increased at 6 h but declined at 12 and 24 h (**Figure 7**). However, viable bacteria in viniferin-treated samples declined after 6 h incubation. Viability of *S. pneumoniae* treated with viniferins **2, 3,** and **8** for 6, 12, and 24 h are shown (**Figure 7**). At 0 h treatment, the cfu counts of the untreated (control) sample and those treated with viniferin **2, 3,** and **8** were approximately similar (4×10^7^ cfu/ml). After 6 h of treatment, the cfu of the untreated sample was elevated and reached 4.30×10^8^ cfu/ml. Following 12 and 24 h of treatment, cfu declined to 4.20×10^7^ and 3.0×10^5^ cfu/ml, respectively. In contrast, the cfu of *S. pneumoniae* treated with viniferins **2**, **3**, and **8** declined in a time-dependent manner. After 6 h treatment with **2** (synthetic), **3**, **8**, and **2** (standard), the cfu counts of *S. pneumoniae* declined to 8.3×10^6^, 1.6×10^7^, 9.0×10^6^, and 8.0×10^6^, respectively. With increasing viniferin **2**, **3**, and **8** treatment time, the viability of *S. pneumoniae* further declined at 12 h (1.6×10^6^, 5.4×10^6^, 5.0×10^6^, and 5.4×10^6^) and 24 h (5.0×10^4^, 4.1×10^5^, 3.0×10^5^, and 5.5×10^4^) (**Figure 7**). Viable bacteria in samples treated with viniferins **2** (synthetic), **3**, **8**, and **2** (standard) decreased by 87–96% at 12 h and 98–99% at 24 h. Among all compounds tested, synthetic and standard ε-viniferin **2** were most effective in killing bacteria (approximately decreased 2.8 log_10_). By definition, a compound that kills >3 log_10_ bacteria following 24 h incubation is considered bactericidal (CLSI, 1999). In this study, ε-viniferin **2** killed a maximum of 2.8 log_10_ bacteria following 24 h incubation and therefore did not qualify for bactericidal activity as per definition.

### Viniferin Increases CV Absorption

**Figure 8:**
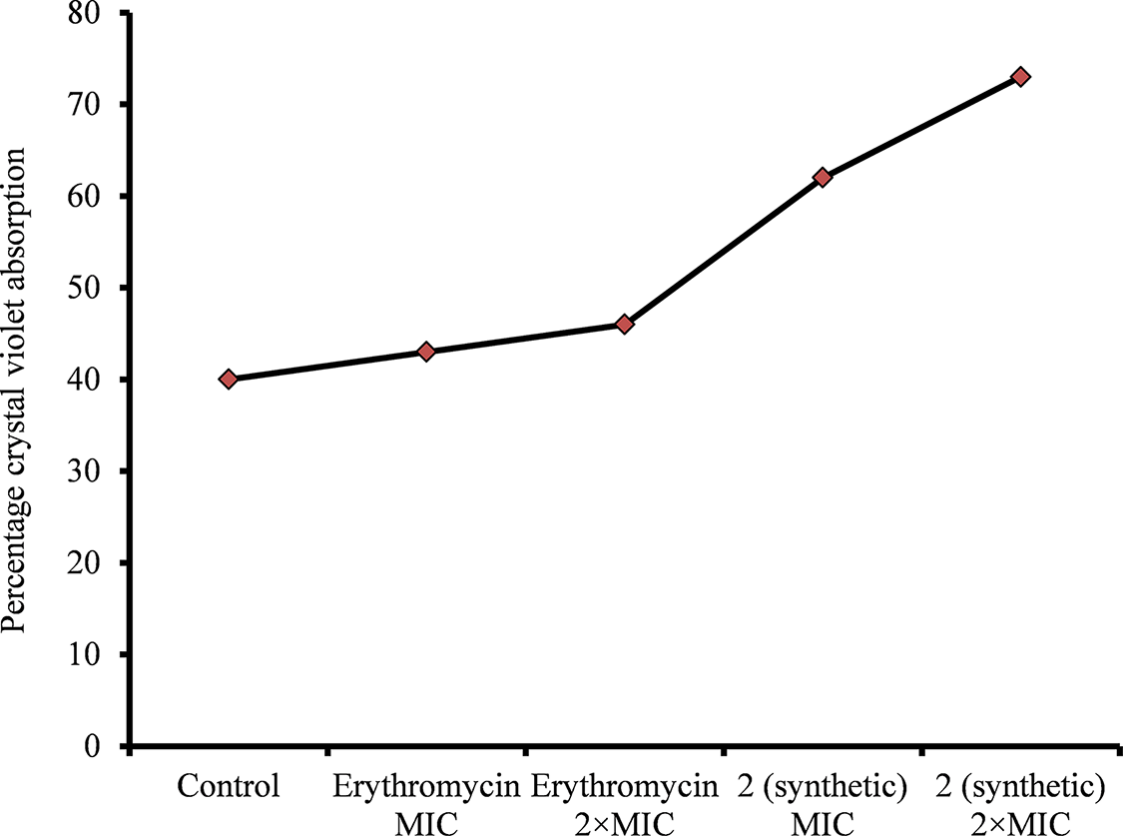
Crystal violet absorption by *S. pneumoniae* D39 treated with ε-viniferin (**2**) or erythromycin. The log-phase *S. pneumoniae* cells were treated with MIC or 2 × MIC of ε-viniferin or erythromycin, and percentage crystal violet absorption was measured as shown in the “Materials and Methods” section.

A CV absorption assay was performed to determine alterations in bacteria cell membrane permeability due to viniferin treatment. The assay demonstrated increased CV absorption by viniferin-treated bacteria, compared with untreated cells (control). CV uptake by untreated bacteria (control) was approximately 40% (**Figure 8**). However, the bacteria treated with ε-viniferin **2** at MIC and 2 × MIC demonstrated 61% and 74% CV uptake, respectively (**Figure 8**). Increased CV absorption by viniferin-treated bacteria indicates that bacterial cell membrane permeability was altered (**Figure 8**). Erythromycin, known for its undetectable effect on cell permeability in non-growing bacteria, was used as a negative control.

### Total Protein, DNA, and RNA Release by Viniferin-Treated Bacteria

**Figure 9:**
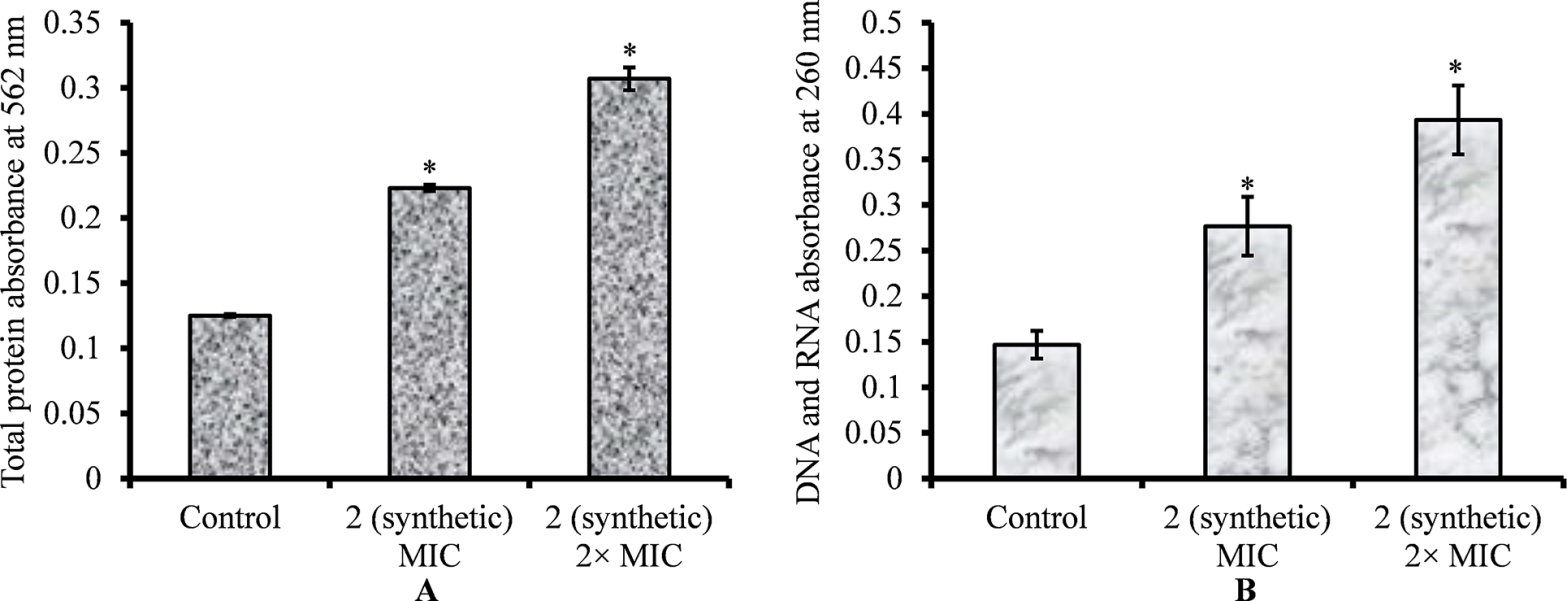
Quantification of total protein and genetic material (DNA and RNA) released by *S. pneumoniae* D39 on viniferin treatment. **(A)** Quantification of total protein released by pneumococci on viniferin treatment (MIC or 2 × MIC), detected by Pierce BCA protein assay. The results are expressed as the absorbance of total proteins at 562 nm. **(B)** Measurement of total DNA and DNA in cell-free supernatant, assessed by detecting absorbance at 260 nm. Error bars represent standard deviations. Statistical significance was calculated using Student’s *t*-test, and *p* < 0.05 was considered significant.

The leakage of cytoplasmic proteins through the cell membrane was examined to evaluate the integrity of pneumococci membrane on viniferin treatment. Protein quantification results showed that total protein release by viniferin-treated bacteria was significantly elevated (P < 0.05) compared to that by control bacteria. Total protein released by bacteria treated with MIC and 2 × MIC viniferin was significantly increased by 43% and 59%, respectively (p < 0.05) (**Figure 9A**).

The leakage of genetic material (DNA and RNA) through the pneumococcal membrane on viniferin treatment was increased. Quantification of DNA and RNA results showed significantly increased (p < 0.05) DNA and RNA in viniferin-treated bacteria (**Figure 9B**). At MIC and 2 × MIC viniferin, DNA and DNA release increased by 46% and 64%, respectively, in comparison to the control (**Figure 9B**).

## Discussion

*S. pneumoniae* causes various infectious diseases and is a leading cause of child morbidity. Approximately 25–60% of healthy children are pneumococci carriers (Korona-Glowniak et al., 2018). Reportedly, biofilms of *S. pneumoniae* are established in the nasopharynx, and planktonic bacteria from biofilms may transit to sterile mucosal tissue, causing Acute Otitis Media (AOM), pneumonia, bacteremia, or local infections (Bogaert et al., 2004). The biofilm resistance paradigm obstructs treatment, resulting in recurrent and recalcitrant infections, which increase recovery time and treatment costs (Costerton et al., 1999; Thomas et al., 2006). Classical antibiotics targeting actively growing bacteria are ineffective due to the dynamic structure of biofilms. Therefore, it is necessary to find new antimicrobials that can effectively control biofilms and prevent the development of antimicrobial resistance (Chopra et al., 2002). One strategy to prevent biofilm-related infections is to inhibit or eradicate initial colonization by bacteria using natural compounds. Reportedly, various phyto-compounds such as 220D-F2 derived from *Rubus ulmifolius*, carvacrol (a constituent of oregano), *Melaleuca alternifolia* (tea tree oil), magnolol, and eugenol are effective against biofilms (Kwieciński et al., 2009; Ma et al., 2012; Quave et al., 2012; van Alphen et al., 2012; Burt et al., 2014; Yadav et al., 2015). We developed a novel method for synthesizing viniferin *via* oxidative coupling with ruthenium chloride and tested its antimicrobial and antibiofilm potential against *S. pneumoniae*.

Different viniferin compounds, ε-viniferin (**2**), (E)-ω-viniferin (**3**), **8**, and **9**, exhibited different MICs for *S. pneumoniae* inhibition. At 20 µm, ε-viniferin **2** completely inhibited bacterial growth, whereas compounds **3**, **8**, and **9** were ineffective. At 40 µm, compounds **3** and **8**, inhibited bacterial growth, but compound **9** did not. These results indicated that ε-viniferin (**2**) is more active compared to compounds **3** and **8**, while **9** was least active. Structural or functional group differences probably contribute to differences in the antimicrobial activity of compounds **2**, **3**, **8**, and **9**. Similarly, differences in antimicrobial activity associated with alterations in structural or functional groups have been reported for eugenol and carvacrol (Cacciatore et al., 2015; da Silva et al., 2018).

*S. pneumoniae* consists of various serotypes, and our results showed that ε-viniferin (**2**) was able to inhibit *S. pneumoniae* serotypes 2, 3, 19A, 19F, and un-encapsulated R6. Serotype 19 is the most prevalent pneumococcus following introduction of the 7-valent pneumococci vaccine (Isturiz et al., 2017; Koutouzis et al., 2017). Treatment of pneumococcal infections caused by antibiotic resistance strains remains a considerable challenge in clinical settings (Korona-Glowniak et al., 2018; Setchanova et al., 2018). Our results showed that ε-viniferin (**2**) effectively inhibited pneumococci resistant to antibiotics (clindamycin, erythromycin, and tetracycline). Altogether, our results indicated that viniferin is equally effective on pneumococci resistant to antibiotics (clindamycin >128, erythromycin >512, and tetracycline 16 µg/ml), as well as serotypes 2, 3, 19A, 19F, and un-encapsulated R6.

Pneumococcal biofilms cause various infections including AOM, pneumonia, bacteremia, and meningitis (Hall-Stoodley et al., 2006; Pichichero et al., 2013; Shak et al., 2013). These biofilms act as bacterial reservoirs from where bacteria can transit in the form of planktonic bacteria to other sterile anatomical sites, causing infections (Hall-Stoodley et al., 2006; Sanchez et al., 2010; Weimer et al., 2010). Therefore, controlling pneumococcal biofilms is important for infection management. Viniferin at MIC prevented biofilm formation in all tested serotypes, including strains resistant to clindamycin >128, erythromycin >512, and tetracycline 16 µg/ml. In contrast, classical aminoglycoside antibiotics accelerate stress in bacteria, resulting in increased biofilm formation at low MICs or sub-MICs (Hoffman et al., 2005). Therefore, an ideal antimicrobial agent should not induce biofilm growth at low concentrations but be effective in preventing biofilm aggregation and eradicating pre-established biofilms. Viniferin shows potential as an ideal antimicrobial agent for pneumococcal biofilm control. Our findings demonstrate that viniferin does not induce biofilm formation at sub-MIC, although it was unable to inhibit pneumococci biofilm formation at sub-MIC. However, it completely inhibited pneumococcal growth under biofilm and planktonic states at MIC. Moreover, viniferin is toxic to bacteria, and the treatment by 2 × MIC viniferin significantly reduced bacterial load by 2.8 log_10_ after 24 h. However, definitions of bactericidal and bacteriostatic indicate that viniferin was not bactericidal (a drug is bacteriostatic if it kills <3 log_10_ bacteria and bactericidal if it kills >3 log_10_ bacteria after 24 h of incubation) (Basri et al., 2014). Similarly, bacteriostatic effects were previously reported for methicillin-resistant *Staphylococcus aureus* (Basri et al., 2014). Reportedly, plant extracts containing viniferin inhibited *in vitro* biofilm growth of Gram-negative bacteria such as *Pseudomonas aeruginosa* and *E. coli*. However, the target of viniferin or underlying mechanisms was not reported (Cho et al., 2013; Lee et al., 2014). In this study, no effects of viniferin on *in vitro* biofilm growth of pneumococci were detected. This implies that viniferin activity is different for Gram-positive and Gram-negative bacteria. The difference between biofilm-inhibiting activities of viniferin in *P. aeruginosa* (Gram-negative bacteria) and *S. pneumoniae* (Gram-positive) may be due to a difference in mechanisms underlying biofilm regulation and biofilm matrix composition (Hall and Mah, 2017). Another reason may be cell membrane differences; the cytoplasmic membrane of Gram-positive bacteria is surrounded by a relatively simple porous cell wall, whereas the cell envelope of Gram-negative bacteria is more complex and relatively impermeable, consisting of an inner cytoplasmic membrane and an outer membrane separated by a peptidoglycan layer (Minnock et al., 2000).

The microbial biofilms are resistant to antibiotics, and the dynamic structure of mature biofilm confers resistance against antimicrobials. Typically, biofilms are surrounded by a self-generated extracellular polymeric matrix (Costerton et al., 1995; Donlan and Costerton, 2002). Reportedly, the charged polymer of the biofilm obstructs drug diffusion through the matrix, thereby limiting drug effects to the surface and leaving bacteria deep inside the biofilm relatively safe (Marsh, 2004). Our results showed that viniferin-treated pre-established biofilms exhibited decreased cfu counts and biofilm biomasses. These results indicated that viniferin may have killed bacteria within the biofilms. SEM analysis of pre-established biofilms treated with viniferin revealed deformed and shrunken cells in biofilms. The bacteria in viniferin-treated biofilms had lost shape, resulting in a shrunken appearance. Confocal microscopy revealed dead cells in viniferin-treated biofilms. CV absorption assay revealed that the viniferin treatment altered bacterial membrane permeability. Leakage of total protein, DNA, and RNA further confirmed that the viniferin treatment disrupted the integrity of the bacterial membrane (Bouyahya et al., 2019). Altogether, SEM analysis; live/dead biofilm staining; CV absorption; and total protein, DNA, and RNA release revealed that viniferin altered bacterial cell permeability and caused cell lysis, indicating that bacteria cell membrane was the target of viniferin. Similar activity was demonstrated by our group in the polyphonic phyto-compounds, eugenol and carvacrol (Devi et al., 2010; Yadav et al., 2013; Yadav et al., 2015; Khan et al., 2017). Classical antibiotics targeting different metabolic pathways of actively growing bacteria are often ineffective against biofilms. Bacteria in biofilm grow slowly, express different genes than planktonic bacteria, and adapt alternate metabolic pathways (Xie et al., 2005; Mascio et al., 2007; Yadav et al., 2012). Biofilms grow slowly in nutrient-depleted conditions, which makes them insensitive to antibiotics (Davies, 2003). The findings of this study and previous reports indicate that viniferin is an important, therapeutic bioactive compound that possesses anti-obesity, anticancer, antimicrobial and antibiofilm properties (Piver et al., 2003; Ohara et al., 2015; Monteillier et al., 2018).

## Conclusion

The current study proposes a simple and short procedure for the synthesis of (±)-ε-viniferin (**2**) and (±)-E-ω-viniferin (**3**) in large quantities. Furthermore, (±)-ε-viniferin (**2**), (±)-E-ω-viniferin (**3**), and compound **8** effectively inhibited *S. pneumoniae* growth and killed bacteria in biofilms. The killing effect of viniferin may due to alteration of cell membrane permeability. Viniferin is known for anti-obesity and anticancer activities, and our study demonstrated its antibacterial and antibiofilm activities against *S. pneumoniae*. Therefore, viniferin and its derivatives show potential as promising candidate molecules for the development of novel antimicrobial agents against pneumococci.

## Data Availability

The raw data supporting the conclusions of this manuscript will be made available by the authors, without undue reservation, to any qualified researcher.

## Author Contributions

MKY and WJC conceived and designed the experiments. MKY, KM, and JNM performed the experiments. MKY, KB, and JNM were in charge of manuscript writing. J-JS, WJC, and MKY were in charge of manuscript review. J-JS, WJC, and S-WC analyzed the data. J-JS, S-WC, and WJC contributed reagents/materials/analysis tools.

## Funding

This research was supported by the Basic Science Research Program of the National Research Foundation (NRF) of Korea funded by Ministry of Education grant (2017R1D1A1B03035306 to MKY). This research was also supported by the Basic Science Research Program through the National Research Foundation of Korea (NRF) funded by the Ministry of Education (NRF-2017R1D1A1B03036116).

## Conflict of Interest Statement

The authors declare that the research was conducted in the absence of any commercial or financial relationships that could be construed as a potential conflict of interest.
